# Medication errors in neonatal intensive care units: a multicenter qualitative study in the Palestinian practice

**DOI:** 10.1186/s12887-022-03379-y

**Published:** 2022-05-30

**Authors:** Ramzi Shawahna, Mohammad Jaber, Rami Said, Khalil Mohammad, Yahya Aker

**Affiliations:** 1 Department of Physiology, Pharmacology and Toxicology, Faculty of Medicine and Health Sciences, An-Najah National University, Nablus, Palestine; 2 An-Najah BioSciences Unit, Centre for Poisons Control, Chemical and Biological Analyses, An-Najah National University, Nablus, Palestine; 3 Department of Medicine, Faculty of Medicine and Health Sciences, An-Najah National University, Nablus, Palestine; 4 An-Najah National University Hospital, Nablus, Palestine

**Keywords:** Medication errors, Newborn, Neonatal intensive care unit, Qualitative analysis

## Abstract

**Background:**

Neonatal intensive care units are high-risk settings where medication errors can occur and cause harm to this fragile segment of patients. This multicenter qualitative study was conducted to describe medication errors that occurred in neonatal intensive care units in Palestine from the perspectives of healthcare providers.

**Methods:**

This exploratory multicenter qualitative study was conducted and reported in adherence to the consolidated criteria for reporting qualitative research checklist. Semi-structured in-depth interviews were conducted with healthcare professionals (4 pediatricians/neonatologists and 11 intensive care unit nurses) who provided care services for patients admitted to neonatal intensive care units in Palestine. An interview schedule guided the semi-structured in-depth interviews. The qualitative interpretive description approach was used to thematically analyze the data.

**Results:**

The total duration of the interviews was 282 min. The healthcare providers described their experiences with 41 different medication errors. These medication errors were categorized under 3 categories and 10 subcategories. Errors that occurred while preparing/diluting/storing medications were related to calculations, using a wrong solvent/diluent, dilution errors, failure to adhere to guidelines while preparing the medication, failure to adhere to storage/packaging guidelines, and failure to adhere to labeling guidelines. Errors that occurred while prescribing/administering medications were related to inappropriate medication for the neonate, using a different administration technique from the one that was intended, and administering a different dose from the one that was intended. Errors that occurred after administering the medications were related to failure to adhere to monitoring guidelines.

**Conclusion:**

In this multicenter study, pediatricians/neonatologists and neonatal intensive care unit nurses described medication errors occurring in intensive care units in Palestine. Medication errors occurred in different stages of the medication process: preparation/dilution/storage, prescription/administration, and monitoring. Further studies are still needed to quantify medication errors occurring in the neonatal intensive care units and investigate if the designed strategies could be effective in minimizing the medication errors.

**Supplementary Information:**

The online version contains supplementary material available at 10.1186/s12887-022-03379-y.

## Introduction

Improving the safety of patients in hospitals is a top priority in all healthcare systems around the world. Reports from different parts of the world have shown that medication errors continue to jeopardize the safety and health of hospital inpatients [1–4]. Medication errors often receive attention from government officials, policymakers in healthcare, professional associations, patient advocacy groups, and the general public. Neonatal intensive care units are high-risk settings where medication errors can occur and cause harm to this fragile segment of patients [3]. Neonates in intensive care units are particularly vulnerable to medication errors because of their immaturity, small body size, need for laborious error-prone dose calculations or dilutions, the severity of their disease condition, and intensity of medications used [3, 5, 6].

In Palestine, patients receive healthcare from 3 main providers: the government, non-governmental organizations, the United Nations Relief and Works Agency for Palestine Refugees in the Near East (UNRWA), and the private sector. Across the country, there are approximately 234 beds in the neonatal intensive care units (192 beds in the West Bank and 42 beds in the Gaza Strip). A recent study reported that governmental and nongovernmental hospitals in Palestine suffered shortages in incubators, ventilators, humidifiers, and ward space were also reported [7]. Additionally, shortages in neonatal nurses, neonatologists, and pediatricians with subspecialties were also reported [7–9]. In the absence of adequate nurse-to-patient ratios, providers of healthcare services in the neonatal intensive care units are often overwhelmed with their daily workloads. Moreover, healthcare providers in hospitals often report receiving insufficient guidelines and training relevant to the safe prescribing, preparation, administration, and monitoring of medications [10–13]. Together, these working conditions increase the likelihood of medication errors in neonatal intensive care units. Although medication error incidence reports are available, healthcare providers described the reporting and surveillance system as ineffective [10, 12]. In the current practice, healthcare providers file a medication error incident report themselves in case of a confirmed medication error that caused serious harm to the patient. Medication errors that did not cause serious harm and near-misses are seldom reported.

In neonatal intensive care units, medications are often prescribed by the attending pediatrician/neontologist. Medications are almost always prepared and administered by the neonatal intensive care unit nurses. Due to shortages of staff, double-check standards for medication preparation and administration are not routinely used in the neonatal intensive care units. Medication errors can occur at any stage in the medication process such as preparation/dilution, packaging, prescribing, dispensing, administration, and monitoring [6]. Previous studies have shown that medication errors occurred in neonatal intensive care units in Qatar, Iran, Saudi Arabia, and Brazil [1, 6, 14, 15]. A recent systematic review reported medication error rates in the range of 4 to 35.1 per 1,000 patient-days in neonatal intensive care units and from 5.5 to 77.9 per 100 medication orders [3]. Dosing errors were the most commonly reported medication errors in neonatal intensive care units. Of all medical errors reported in neonatal intensive care units, medication errors accounted for 47.2% of all errors [16]. Medication errors that cause harm to the patients are eightfold more likely to occur in neonatal intensive care units compared to those occurring in adult intensive care units [5].

It is noteworthy mentioning that the majority of the studies that reported on medication errors in neonatal intensive care units were quantitative in nature. Therefore, medication error situations have not been described in sufficient details. Qualitative research methods are commonly used as powerful tools in describing complex phenomena, shedding light on the experiences of the study participants, exploring views of key actors and stakeholders in certain phenomena, developing new theories, and seeking interpretations [17, 18]. Additionally, qualitative and quantitative approaches can be used in sequence, tandem, and/or can be complementary to each other.

Little was reported on the incidence of medication errors in neonatal intensive care units in Palestine and medication errors have not been described before. In a previous study, the safety culture in different neonatal intensive care units in Palestine was described as suboptimal [8]. Understanding medication errors occurring in neonatal intensive care units might help design strategies to minimize these errors and improve the safety of patients and healthcare delivery in neonatal intensive care units. Therefore, this multicenter qualitative study aimed to describe medication errors that occurred in neonatal intensive care units in Palestine from the perspectives of healthcare providers.

## Methods

### Study design

This exploratory multicenter qualitative study was conducted and reported in adherence to the consolidated criteria for reporting qualitative research (COREQ) checklist. Adherence to the COREQ checklist is provided in Supplementary Table S1. Semi-structured in-depth interviews were conducted with healthcare professionals who provided care services for patients admitted to 4 main neonatal intensive care units in Palestine. An interview schedule guided the semi-structured in-depth interviews.

### Sample size and sampling method

In this qualitative study, thematic saturation was used to determine the endpoint of the interviews. Informed by previous qualitative studies that were conducted with healthcare providers, thematic saturation was expected to be achieved in about 5 h of interview time [13, 19–21]. Assuming an average interview duration of 30 min, interviews with 10 healthcare providers were expected to achieve thematic saturation in this study.

A purposive sampling technique was used to identify, invite, and recruit the interviewees. Personal contacts in the field were used to approach and invite the potential interviewees who were pediatricians/neonatologists and intensive care unit nurses who were the providers of care services to patients admitted to the 4 main neonatal intensive care units in Palestine. The healthcare providers were invited and recruited when they satisfied the following inclusion criteria: 1) being licensed to practice medicine or nursing in Palestine, 2) practicing in a hospitalized patient setting in Palestine, 3) providing direct care using medications in a neonatal intensive care unit in Palestine, and 4) having at least 5 years of practical experience in a neonatal intensive care unit in Palestine. Healthcare providers who did not meet the inclusion criteria and those who did not agree to participate in a recorded qualitative interview were excluded from this study.

### Data collection

The healthcare providers were interviewed by three male 6th-year medical students (RS, KM, YA) who were trained to conduct semi-structured in-depth interviews by the principal investigator (RS) who had extensive experience in conducting interviews and qualitative studies. Throughout their clinical training, the three students received formal and supervised training on how to conduct interviews and communicate with patients, nurses, physicians, and other healthcare providers. The interviews in this study were conducted with the help of an interview schedule that was developed specifically for this study. The interview schedule was developed to contain open-ended questions to allow the interviewees to provide an elaborated description of the medication errors. The interviewers also used prompts to clarify answers and descriptions provided by the healthcare providers during the interviews. The interview guide was reviewed by 5 healthcare providers to ensure face validity and ability of the questions to collect adequate descriptions of medication errors. The reviewers were asked to rate each question for clarity, relevance, and suitability on a scale of 1–5 (1 = not clear/relevant/suitable at all, 5 = very clear/relevant/suitable). The questions that were rated as clear/relevant/suitable or very clear/relevant/suitable were included in the interview guide. Conflicting views were discussed and resolved through consensus.

The healthcare providers were interviewed in a quiet place in the hospitals. The interview guide collected the sociodemographic and practice characteristics of the healthcare providers like gender, specialty, type of hospital, and number of years in practice. The interviewees were asked to self-report the approximate number of neonatal patients they care for per shift. The interviewees were also asked to provide an approximate number of patients admitted to each neonatal intensive care unit per month and reasons for admission of patients to the neonatal intensive care unit. The interviewees were asked to indicate if a functional system for reporting medication errors and near-misses existed in the neonatal intensive care units, if they believed that neonatal intensive care units were high-risk settings, if they believed medication errors in neonatal intensive care units were more likely compared to adult settings, and to provide reasons why they believed so. The healthcare providers were then asked to describe medication errors they experienced in the neonatal intensive care units. The interview guide is provided in Supplementary Table S2. The interviews were audio-recorded and the interviewers took field notes.

### Data analysis

All recordings were transcribed verbatim before analysis. The qualitative interpretive description approach was used to thematically analyze the data [22–25]. The qualitative interpretive description approach is powerful in promoting the recognition of categories and subcategories in cases that involve diverse and complex experiences such as medication errors [26]. Additionally, the Leuven Qualitative Analysis Guide was used to help identify categories and subcategories [27]. The researchers independently read and familiarized themselves with the transcripts (the transcripts were read 34 times). The researchers independently identified the important statements, formulated their meanings, and clustered them into major categories. The researchers met and developed a detailed description of the major categories and subcategories, and condensed the medication errors into the major categories and subcategories. Finally, the researchers verified if all medication errors were accurately captured and categorized under the major categories and subcategories. To protect participant identity, who could may have made the medication error, nurses, physicians, or any other healthcare provider were coded as “a healthcare provider”.

Bracketing was used to maintain the trustworthiness and credibility of the analysis. Throughout the study, the researchers made all efforts to keep their personal, knowledge, experiences, and expectations aside to avoid any preconception bias [28, 29]. To ensure dependability, the researchers kept and reviewed audit trails of the analytical decisions. Repeated readings of the transcripts, regular discussion between the researchers, consensus to solve conflicting decisions, and triangulation of the qualitative data from each interview script were used to ensure accurate sorting of the error situations into the major categories and subcategories [30].

## HE

All aspects of the study protocol and ethics were approved by the Institutional Review Board (IRB) of An-Najah National University. Approvals were also obtained from the administration, ethics, and/or research committees of the 4 hospitals. The study was conducted in adherence to the principles of scientific and medical research included in the Declaration of Helsinki. All participants provided written informed consent before they took part in this study.

## Results

Fifteen healthcare providers participated in the interviews. The total duration of the interviews was 282 min (range: 15–25 min).

### Characteristics of the interviewees

Of the interviewees, 4 (26.7%) were pediatricians/neonatologists and 11 (73.3%) were neonatal intensive care nurses. For the pediatricians/neonatologists, the median number of years in practice was 22.5 (20.5, 25.8) years and the median number of years in practice for the neonatal intensive care nurses was 11 (8.5, 12.5) years. The median number of neonatal patients care for by the pediatricians/neonatologists per day was 14.5 (13.0, 15.8) patients and the median number of neonatal patients care for by the nurses per day was 6.0 (6.0, 7.0) patients. Of the interviewees, 10 (66.7%) were employed by governmental hospitals. The characteristics of the interviewees stratified by the type of hospital are provided in Table 1.

**Table 1:** Characteristics of the interviewees (*n* = *15*)

Characteristic	Type of hospital	
	**Governmental**	**Private**
**Gender**		
Male	5	3
Female	5	2
**Profession**		
Pediatrician/neonatologist	3	1
Neonatal intensive care nurse	7	4
**Number of years in practice**		
< 10	3	2
≥ 10	7	3
**Self-reported approximate number of neonatal patients care for per shift**		
< 8	5	1
≥ 8	5	4

### Characteristics of the patients admitted to the neonatal intensive care units

The interviewees stated that there were about 250 to 300 new admissions to each neonatal intensive care unit per month. The majority of the patients admitted to the neonatal intensive care units were preterm or extremely preterm with low or very low birth bodyweight. The other common reasons for admission included preterm neonates who developed hyaline membrane disease/acute respiratory distress syndrome, transient tachypnea of the newborn, sepsis, congenital diaphragmatic hernia, and/or jaundice.

### Experiences of the interviewees with medication errors in the neonatal intensive care unit

The interviewees stated that not all neonatal intensive care units have a functional system for reporting medication errors and near-misses. One of the interviewees stated:

“…a fully functional system for reporting medication errors and near-misses does not exist in our neonatal intensive care unit. We might file an incidence report when a medication error caused a serious harm to the patient.”

The healthcare providers who were interviewed in this study agreed that neonatal intensive care units were high-risk settings and medication errors were more likely compared to adult settings because of the need for laborious error-prone medication dose calculations/dilutions. The healthcare providers also stated that neonates were more vulnerable to harm from medication errors compared to adults because of their immaturity, small body size, and disease conditions. One of the interviewees stated:

“…..there is no doubt that medication errors are more likely in neonatal intensive care settings. Patients are immature, have a small body size, and are fragile.”

In this study, the healthcare providers described experiencing 41 different medication errors. These medication errors were categorized under 3 main categories and 10 subcategories.

### Errors that occurred while preparing/diluting/storing a medication

In this study, the interviewees described 24 medication errors that occurred while preparing/diluting/storing medications. Of those 24 errors, 3 (12.5%) were related to calculations, 3 (12.5%) were related to using a wrong solvent/diluent, 3 (12.5%) were dilution errors, 6 (25.0%) were related to failure to adhere to guidelines while preparing a medication, 4 (16.7%) were related to failure to adhere to storage/packaging guidelines, and 5 (20.8%) were related to failure to adhere to labeling guidelines. Details of these medication errors are shown in Table 1.

### Errors that occurred while prescribing/administering a medication

The interviewees described 15 medication errors that occurred while prescribing/administering a medication. Of those errors, 5 were related to inappropriate medication for the neonate, 5 were related to a different administration technique from the one that was intended, and 5 were related to a different dose from the one that was intended. Details of these medication errors are shown in Table 2.

**Table 2:** Summary of the medication errors that occurred in neonatal intensive care units that were experienced by the interviewees

#	Category	Medication errors
	**Errors that occurred while preparing/diluting/storing a medication**	
1	Calculation errors	A healthcare provider failed to correctly calculate a dose of a medication for a neonate and the final preparation contained an overdose/underdose
2		A healthcare provider failed to verify an ambiguous medication preparation order and prepared a dose of a medication for a neonate using cubic centimeters (cc)/milliliters (mL) when milligrams (mg) were intended
3		A healthcare provider failed to verify the correct weight of the neonate and calculated a dose of a medication based on an incorrect weight. The dose resulted in an overdose/underdose
4	Using a wrong solvent/diluent	A healthcare provider failed to adhere to the preparation guidelines and used the wrong solvent instead of distilled water while preparing a dose of potassium chloride for a neonate
5		A healthcare provider withdrew sodium bicarbonate instead of amino acids from a look-alike ampule while preparing a dose for a neonate
6		A healthcare provider failed to adhere to the preparation guidelines and used normal saline instead of dextrose while preparing a dose of ertapenem for a neonate
7	Dilution errors	A healthcare provider failed to adhere to the preparation guidelines and diluted a dose of adrenaline that was intended for endotracheal administration for a neonate
8		A healthcare provider failed to adhere to the preparation guidelines and did not dilute an intravenous dose of aminophylline that was intended for a neonate
9		A healthcare provider failed to adhere to the preparation guidelines and used an excessive amount of the diluent which resulted in a subtherapeutic dose that was intended for a neonate
10	Failure to adhere to guidelines while preparing a medication	A healthcare provider failed to check the expiry dates of the ingredients used to prepare a dose of a medication that was intended for a neonate
11		A healthcare provider failed to check a broken vial/ampoule that contained pieces of glass before using it in preparing a dose of a medication that was intended for a neonate
12		A healthcare provider failed to adhere to the guidelines and did not use different syringes and needles while preparing doses of different medications that were intended for neonates
13		A healthcare provider failed to adhere to the guidelines and prepared a dose of medication that required strict aseptic techniques in a contaminated area
14		A healthcare provider failed to adhere to the guidelines and did not completely dissolve the ingredients leaving precipitates in an intravenous preparation that was intended for a neonate
15		A healthcare provider failed to calibrate the balance used to prepare a dose of a medication for a neonate and the final preparation contained an overdose/underdose
16	Failure to adhere to storage/packaging guidelines	A healthcare provider failed to adhere to the storage guidelines for a light-sensitive medication and the medication was exposed to light for a significantly long time
17		A healthcare provider failed to adhere to the storage guidelines and stored a medication in a humid environment while the instructions dictated that the medication should be stored in a dry place
18		A healthcare provider failed to adhere to the storage guidelines and stored a medication at room temperature while the instructions dictated that the medication should be stored in a refrigerator
19		A healthcare provider failed to adhere to the guidelines and packed doses of two different medications using identical packages
20	Failure to adhere to labeling guidelines	A healthcare provider failed to adhere to the guidelines and did label a prepared dose of the medication with the name of the neonate as in the wristband
21		A healthcare provider failed to adhere to the guidelines and did label a prepared dose of the medication with the name of the medication
22		A healthcare provider failed to adhere to the guidelines and did label a prepared dose of the medication with the dose
23		A healthcare provider failed to adhere to the guidelines and did label the dose of the medication with the route of administration
24		A healthcare provider failed to adhere to the guidelines and did label a prepared dose of the medication with the date of preparation/expiry
	**Errors that occurred while prescribing/administering a medication**	
1	The prescribed/administered medication was not appropriate for the neonate	A healthcare provider failed to check the wristband of a neonate and administered a dose of a medication that was intended for another neonate
2		A healthcare provider did not verify the expiry date of the medication and administered a dose of an expired medication to a neonate
3		A healthcare provider failed to check the patient’s allergy notes and prescribed/administered a dose of vancomycin when the notes indicated that the neonate had an allergy to vancomycin
4		A healthcare provider administered two medications for a neonate that were known to have a significant drug-drug interaction
5		A healthcare provider failed to verify an ambiguous medication order and administered a different medication for a neonate from the one that was intended (e.g., ibuprofen when paracetamol/acetaminophen was intended)
6	The administration technique was different from the one that was intended	A healthcare provider failed to verify an ambiguous medication order and administered a dose of a medication for a neonate using a route of administration that was different from the one that was intended (e.g., intravenous prednisolone when inhaled was intended, oral when intravenous was intended, and intramuscular when intravenous was intended)
7		A healthcare provider failed to adhere to the administration guidelines and administered a dose of a medication for a neonate using rapid intravenous push when the instructions dictated that the dose had to be administered slowly over a longer period (e.g., fentanyl, potassium chloride, and gentamycin)
8		A healthcare provider failed to insert the cannula correctly and administered a dose of a medication that is known to cause extravasation (e.g., total parenteral nutrition, potassium, calcium, bicarbonate, and high concentration dextrose)
9		A healthcare provider failed to adhere to the administration instructions and administered a dose of a medication for a neonate over a shorter/longer period from the one that was intended (e.g., administration for 10 min when administration for 30 min was intended)
10		A healthcare provider failed to verify if the cannula was open and started administering the medication while the cannula was closed
11	The administered dose was different from the one that was intended	A healthcare provider failed to adhere to the prescription/administration instructions and forgot to administer the medication to the neonate
12		A healthcare provider failed to verify an ambiguous medication order for a neonate and administered a dose of 10 mg morphine when 1 mg was intended
13		A healthcare provider failed to verify an ambiguous medication order for a neonate and administered the medication at a frequency that was different from the one that was intended (e.g., every 3 h instead of every 6 h or every 6 h instead of every 3 h)
14		A healthcare provider failed to program the intended infusion rate on an infusor or used the infusion rate that was programmed for the previous medication
15		A healthcare provider failed to adhere to the prescription/administration instructions and administered the calculated maintenance dose instead of the loading dose for a neonate
	**Errors that occurred after administering a medication (monitoring errors)**	
1	Failure to adhere to monitoring guidelines	A healthcare provider failed to adhere to the monitoring guidelines and failed to monitor renal function after a dose of a medication that caused nephrotoxicity in a neonate (e.g., vancomycin)
2		A healthcare provider failed to adhere to the monitoring guidelines and failed to monitor heart rate after administering a dose of a medication to a neonate that caused cardiac arrhythmias (e.g., calcium gluconate, potassium chloride, and fentanyl)

### Errors that occurred after administering a medication (monitoring errors)

The interviewees described 2 monitoring error incidents that occurred after administering medications. These errors were related to failure to adhere to monitoring guidelines. Details of these medication errors are shown in Table 2.

## Discussion

Medication errors in neonatal intensive care units can result in significant harm to this subsegment of fragile patients. This multicenter qualitative study was conducted to describe medication errors in 4 main neonatal intensive care units in Palestine. To the best of our knowledge, this is the first description of medication errors that occurred in neonatal intensive care units in Palestine. A total of 41 different medication errors were described by the healthcare providers. The findings of this study could be informative to policymakers in healthcare authorities, professional organizations, patient advocacy groups, and other stakeholders who might need to take action and design strategies to minimize medication errors in neonatal intensive care units.

Preterm/extremely preterm with low/very low birth bodyweight, development of hyaline membrane disease/acute respiratory distress syndrome, transient tachypnea of the newborn, sepsis, congenital diaphragmatic hernia, and/or jaundice were the common reasons for admitting neonates to the neonatal intensive care units in Palestine. These findings were consistent with those reported on the common reasons for admitting neonates to neonatal intensive care units elsewhere [31, 32]. Although the majority of the healthcare providers considered neonatal intensive care units as high-risk settings, a functional system for reporting medication errors and near-misses was not present in all neonatal intensive care units in Palestine. It is universally accepted that reporting medication errors and near-misses is of great importance [33]. It has been argued that quantitative, as well as qualitative analysis of root causes of medication errors, can aid in designing strategies to minimize these errors [34, 35]. The findings of this study may add to the existing literature by providing a qualitative description of medication errors by healthcare providers who experienced these medication errors.

In this study, the healthcare providers described medication errors that occurred while preparing/diluting/storing, prescribing/administering, and monitoring administered medications. The findings of this qualitative study were consistent with those that previously reported medication errors during the different stages of the medication process [6, 36]. Because of their small body size, doses of medications for neonates often need to be individualized. This would often require laborious error-prone dose calculations/dilutions which may result in overdoses that lead to toxicity or underdoses that may prolong the stay of the neonate in the intensive care unit [3, 5, 6, 34]. Overdoses and subtherapeutic doses of some medications can lead to serious adverse effects, delay recovery, increase the length of hospitalization, and/or even can result in the death of the patients [37, 38]. Review of the dose calculations, preparation, dilution, and administration steps by a second healthcare provider (double-check) and using a computerized program to calculate the individualized doses might reduce calculation errors [38]. Using the wrong solvent/diluent and failure to adhere to guidelines while preparing the medication were reported by the healthcare providers in this study. The findings of this study were consistent with those reported in previous studies in neonatal intensive care units [3, 5, 6, 36]. The use of look-alike ampoules/vials and lack of adherence to basic preparation guidelines increased the incidence of medication errors. Manufacturers need to consider distinguishing ampoules/vials using distinct colors/sizes/shapes/designs and policymakers/managers should consider training/educating healthcare providers on the importance of adhering to the preparation guidelines. These measures might reduce the incidence of these errors in neonatal intensive care units [5, 39]. Moreover, the production of age-appropriate dosage units for neonates by the pharmaceutical industry can reduce the need for extemporaneous preparation/dilution of medications for this segment of patients and, thus, can reduce the incidence of these medication errors [40]. In this study, the healthcare providers reported that failure to adhere to storage/packaging guidelines resulted in medication errors in the neonatal intensive care units. The findings of this study indicated that healthcare providers need to be trained/educated on the importance of adherence to storage/packaging guidelines to reduce the incidence of these errors [35, 39]. Mislabeling was also reported by the healthcare providers who were interviewed in this study. Complete and accurate labels of prepared medications are important to avoid confusion and situations leading to harmful medication errors [10, 11, 35]. In this study, 15 prescription/administration medication errors were reported by the healthcare providers. Error situations were related to prescribing/administering an inappropriate medication to the patient, using an erroneous administration technique, or a wrong dose. These situations were formally considered medication errors that needed to be eliminated by panels of healthcare providers [12]. Some medications need to be monitored following administration. Adequate monitoring can reduce the incidence of adverse effects that can be associated with these medications. In this study, lack of adequate monitoring resulted in harmful adverse effects. These findings were consistent with those that reported harmful medication errors among patients admitted to neonatal intensive care units [3, 6, 16].

Decision-makers in healthcare authorities and other stakeholders should consider designing strategies to minimize medication errors occurring in neonatal intensive care units and improve the health outcomes of neonates. These interventions might include educational and training sessions to improve awareness, adherence to guidelines, and numeracy skills of the healthcare providers in neonatal intensive care units [5, 39, 41, 42]. Additionally, the use of computers, robots, bar-codes, double-check standards, improving storage layout, and labeling of vials/ampoules might also help minimize medication errors that occur while preparing/diluting/storing the medication in neonatal intensive care units [16]. It has been suggested that using age-appropriate pre-filled and pre-labeled doses can eliminate the need for the preparation or dilution of medication doses [40, 43]. This would ultimately minimize medication errors. Employing a clinical pharmacist as a member of the healthcare team might minimize medication errors in the neonatal intensive care units [44, 45]. Clinical pharmacists are experts in medications and can help optimize doses of medications, monitor their effects, and screen for adverse effects.

### Strengths and limitations of the study

This multicenter explorative study had several strengths. In this study, medication errors in neonatal intensive care units were reported for the first time. Understanding these medication errors can help stakeholders design appropriate measures to minimize these errors. Second, the healthcare providers who were interviewed in this study were providers of care services in the main neonatal intensive care units in Palestine. Multicenter recruitment of the healthcare providers should have allowed a description of the main medication error situations that occurred in these centers. Third, although pediatricians/neonatologists were included, the majority of the healthcare providers who were interviewed in this study were intensive care unit nurses. Recruitment of nurses was purposive because intensive care unit nurses provide the majority of healthcare services to neonates admitted to these units. The healthcare services provided by intensive care unit nurses include calculation of doses, preparation/dilution of medications, administration of medications, monitoring therapeutic effects, and screening for adverse effects. Fourth, the interviewees were diversified in terms of gender, specialty, type of hospital, length of practical experience, and the number of neonates cared for. This diversity should have added strength and rigor to the qualitative data generated in this study.

On the other hand, the findings of this multicenter study should be interpreted considering some limitations. First, a qualitative approach was used in this study. Qualitative data are merely opinions and views of the interviewees. Although qualitative approaches are powerful in describing medication errors, a quantitative approach by counting the number of errors in each error category should have added another dimension to the findings of this study. In this study, we could have attempted to quantify the medication errors that occurred in neonatal intensive care units. However, in the absence of an effective error reporting and surveillance systems, direct observation would be the only alternative. Second, data generated in a qualitative are prone to bias by the opinions of the interviewers. Although bias cannot be completely ruled out, audio recordings of the interviews, bracketing, reviews of audit trails, repeated readings of the transcripts, regular discussion between the researchers, using consensus to solve conflicting decisions, and triangulation of the qualitative data should have improved rigor, trustworthiness, and credibility of the data generated in this study. Third, parents/legal guardians of neonates were not interviewed in this study. Identifying parents/legal guardians of neonates who have experienced medication errors could have ensured the inclusion of their description of their experience with medication errors in neonatal intensive care units. Finally, we did not classify the medication errors based on the severity of their outcomes or harm caused to the patient. Such classification should have been more informative. Future studies should consider categorizing medication error situations by the harm caused to the patient.

## Conclusion

In this multicenter study, pediatricians/neonatologists and neonatal intensive care unit nurses described medication errors occurring in intensive care units in Palestine. Medication errors occurred in different stages of the medication process: preparation/dilution/storage, prescription/administration, and monitoring. Further studies are still needed to quantify medication errors occurring in the neonatal intensive care units and investigate if the designed strategies could be effective in minimizing the medication errors.

## Supplementary Information

**Additional file 1: Supplementary Table S1. **Adherence to COnsolidated criteriafor REporting Qualitative research (COREQ)Checklist [1]. **Supplementary Table S2. **Interview guide. 

## Data Availability

All data analyzed during this study are included in the results section of this manuscript and the supplementary materials.
