# Supplementary material for: Medication errors in neonatal intensive care units: a multicenter qualitative study in the Palestinian practice
Source: BMC Pediatr. 2022 May 30;22:317. doi: 10.1186/s12887-022-03379-y (PMC9150293; doi:10.1186/s12887-022-03379-y)
Supplement: Supplementary file 1 — Additional file 1: Supplementary Table S1. Adherence to COnsolidated criteriafor REporting Qualitative research (COREQ)Checklist [1]. Supplementary Table S2. Interview guide. [file 12887_2022_3379_MOESM1_ESM.docx]

**Supplementary materials for the manuscript**

**Medication errors in neonatal intensive care units: A multicenter qualitative study in the Palestinian practice**

Ramzi Shawahna^1,2*^, Mohammad Jaber^3,4^, Rami Said^3^, Khalil Mohammad^3^, Yahya Aker^3^

^1^Department of Physiology, Pharmacology and Toxicology, Faculty of Medicine and Health Sciences, An-Najah National University, Nablus, Palestine

^2^An-Najah BioSciences Unit, Centre for Poisons Control, Chemical and Biological Analyses, An-Najah National University, Nablus, Palestine

^3^Department of Medicine, Faculty of Medicine and Health Sciences, An-Najah National University, Nablus, Palestine

^4^An-Najah National University Hospital, An-Najah National University, Nablus, Palestine

**^*^Correspondence:**

Ramzi Shawahna, PhD, Department of Physiology, Pharmacology and Toxicology, Faculty of Medicine & Health Sciences, New Campus, Building: 19, Office: 1340, An-Najah National University, P.O. Box 7, Nablus, Palestine

Phone: + (970) 923 45113 ext 2772

Phone: + (970) 92349739

Email: [ramzi_shawahna@hotmail.com](mailto:ramzi_shawahna@hotmail.com)

**Supplementary Table S1:** Adherence to COnsolidated criteria for REporting Qualitative research (COREQ) Checklist [[1](#_ENREF_1)]

| **#** | **Topic** | **Guide Questions/Description** | **Page/Line # in the manuscript** |
| --- | --- | --- | --- |
|  | **Domain 1: Research team and reﬂexivity** |  |  |
|  | *Personal characteristics* |  |  |
| 1 | Interviewer/facilitator | Which author/s conducted the interview or focus group? | Under data collection section |
| 2 | Credentials | What were the researcher’s credentials? E.g. PhD, MD | Under data collection section |
| 3 | Occupation | What was their occupation at the time of the study? | Under data collection section |
| 4 | Gender | Was the researcher male or female? | Under data collection section |
| 5 | Experience and training | What experience or training did the researcher have? | Under data collection section |
|  | *Relationship with participants* |  |  |
| 6 | Relationship established | Was a relationship established prior to study commencement? | Under Sample size and sampling method |
| 7 | Participant knowledge of the interviewer | What did the participants know about the researcher? e.g. personal goals, reasons for doing the research | Under Sample size and sampling method |
| 8 | Interviewer characteristics | What characteristics were reported about the inter viewer/facilitator? e.g. Bias, assumptions, reasons and interests in the research topic | Under data collection section |
|  | **Domain 2: Study design** |  |  |
|  | *Theoretical framework* |  |  |
| 9 | Methodological orientation and Theory | What methodological orientation was stated to underpin the study? e.g. grounded theory, discourse analysis, ethnography, phenomenology, content analysis | Under Data analysis section |
|  | *Participant selection* |  |  |
| 10 | Sampling | How were participants selected? e.g. purposive, convenience, consecutive, snowball | Under Sample size and sampling method |
| 11 | Method of approach | How were participants approached? e.g. face-to-face, telephone, mail, email | Under Sample size and sampling method |
| 12 | Sample size | How many participants were in the study? | Under Sample size and sampling method |
| 13 | Non-participation | How many people refused to participate or dropped out? Reasons? | N/A |
|  | *Setting* |  |  |
| 14 | Setting of data collection | Where was the data collected? e.g. home, clinic, workplace | Under Characteristics of the interviewees |
| 15 | Presence of non-participants | Was anyone else present besides the participants and researchers? | N/A |
| 16 | Description of sample | What are the important characteristics of the sample? e.g. demographic data, date | Under Characteristics of the interviewees |
|  | *Data collection* | | |
| 17 | Interview guide | Were questions, prompts, guides provided by the authors? Was it pilot tested? | Yes, under Data collection section (supplementary materials) |
| 18 | Repeat interviews | Were repeat interviews carried out? If yes, how many? | no |
| 19 | Audio/visual recording | Did the research use audio or visual recording to collect the data? | Under Data collection section |
| 20 | Field notes | Were ﬁeld notes made during and/or after the interview or focus group? | N/A |
| 21 | Duration | What was the duration of the inter views or focus group? | Under the Results section |
| 22 | Data saturation | Was data saturation discussed? | Under Sample size and sampling method |
| 23 | Transcripts returned | Were transcripts returned to participants for comment and/or correction? | Under Data collection and Data analysis section |
|  | **Domain 3: analysis and ﬁndings** |  |  |
|  | *Data analysis* | | |
| 24 | Number of data coders | How many data coders coded the data? | Under Data analysis |
| 25 | Description of the coding tree | Did authors provide a description of the coding tree? | Under Data analysis |
| 26 | Derivation of themes | Were themes identiﬁed in advance or derived from the data? | Under Data analysis |
| 27 | Software | What software, if applicable, was used to manage the data? | N/A |
| 28 | Participant checking | Did participants provide feedback on the ﬁndings? | Under Data analysis |
|  | *Reporting* |  |  |
| 29 | Quotations presented | Were participant quotations presented to illustrate the themes/ﬁndings? Was each quotation identiﬁed? e.g. participant number | Yes under the results section |
| 30 | Data and ﬁndings consistent | Was there consistency between the data presented and the ﬁndings? | Yes under discussion section |
| 31 | Clarity of major themes | Were major themes clearly presented in the ﬁndings? | Yes under the results section |
| 32 | Clarity of minor themes | Is there a description of diverse cases or discussion of minor themes? | Yes under the results section |

**Supplementary Table S2:**

**Interview guide**

| ·         Gender: □ Male □ Female |
| --- |
| ·         Specialty: □ Pediatrician/neontologist □ Intensive care unit nurse |
| ·         Type of hospital: □ Governmental □ Private |
| ·         Number of years in practice: ………………. |
| ·         Approximately, how many neonates do you provide care for in a typical day? …………….. |
| ·         In the neonatal care unit where you practice, approximately, how many neonates are admitted per month? ………………………. |
| ·         What are the common health conditions that necessitated admission of the neonates in the neonatal care unit where you practice? |
| ·         Could you please tell us about the error/near-miss reporting systems in the neonatal intensive care unit where you practice? |
| ·         Do you think that neonatal intensive care units are high-risk settings? Why? |
| ·         Do you think that medication errors in neonatal intensive care units are more likely compared to adult settings? Why? |
| ·         Could you tell us about the medication errors/situations/incidents that you have experienced or witnessed in neonatal intensive care units? Could you please elaborate? |
